# Dissociation of Caloric and Video Head Impulse Tests in Patients With Delayed Endolymphatic Hydrops

**DOI:** 10.3389/fneur.2020.00362

**Published:** 2020-05-12

**Authors:** Yangming Leng, Bo Liu

**Affiliations:** Department of Otorhinolaryngology, Union Hospital, Tongji Medical College, Huazhong University of Science and Technology, Wuhan, China

**Keywords:** delayed endolymphatic hydrops, caloric test, video head impulse test, VOR, semicircular canal

## Abstract

Delayed endolymphatic hydrops (DEH) represents a rare clinical entity characterized by intermittent vertigo attacks mimicking those of Ménière's disease (MD) in a patient with a prior sensorineural hearing loss. Some vestibular tests have been employed in patients with DEH. These tests provide useful diagnostic information and facilitate clinical decision-making. Here, we retrospectively studied the features of video head impulse test (vHIT) and examined its relationship with caloric test used in DEH patients. Included in this study were 17 patients with ipsilateral DEH and 2 with contralateral DEH. Among them, 73.7% (14/19) showed abnormal caloric test response (76.5% in ipsilateral DEH and 50% in contralateral DEH). Meanwhile, only 15.8% (3/19) of patients yielded abnormal horizontal vHIT results (11.8% in ipsilateral DEH and 50% in contralateral DEH). Abnormal caloric response in the presence of a preserved vHIT was common in DEH patients, especially those with ipsilateral DEH. This dissociation might be a distinctive pattern of vestibular deficit in DEH.

## Introduction

Vestibular test is designed to assess the status of the vestibular system objectively and quantitatively. Over the last decade, vestibular test has evolved greatly, and the horizontal and vertical semicircular canals (SCC) as well as the otolith organs can be examined separately (1). Traditionally, the caloric test has been used for the assessment of the vestibulo-ocular reflex (VOR) function of horizontal SCC by using a non-physiological stimulus in a frequency ranging 0.002–0.004 Hz (2). Video head impulse test (vHIT) extends the testing range to physiological high frequency (5–7 Hz) (3). The relationship between these two VOR tests has been investigated in multiple vestibular disorders, such as Ménière's disease (MD), vestibular neuritis, vestibular migraine, benign paroxysmal positioning vertigo (BPPV), enlarged vestibular aqueduct (EVA), among others (2, 4, 5). Recently, a dissociation between caloric test and vHIT was found to be common in patients with MD (6, 7), and has therefore been suggested as an instrumental hallmark of MD (5).

Delayed endolymphatic hydrops (DEH), a rare clinical entity, is characterized by episodic vertigo of delayed onset following profound sensorineural hearing loss (SNHL), and its symptoms mimic those of MD (8). Kamei et al. for the first time, reported the association between “juvenile-onset unilateral profound deafness” and delayed onset of vertigo in 1971 (9). In 1973, when investigating the pathological features in a case of profound congenital deafness with delayed-onset vertigo, Schuknecht and Wright presumed that endolymphatic hydrops (ELH) secondary to fibrous obliteration of the vestibular aqueduct might underlie vertigo (10). Nadol et al. and Wolfson and Leiberman also described the unilateral deafness with subsequent vertigo in 1975 (11, 12), and suggested the hydropic change due to obliteration of the vestibular aqueducts could cause this delayed-onset vertigo. Furthermore, Schuknecht proposed a hypothesis postulating that an initial labyrinthine insult leads to total disruption of cochlear but preserves vestibular function, and then results in secondary atrophy or fibrous obliteration of the endolymphatic resorptive system (13). In 1978, Schuknecht coined the term DEH to describe the delayed development of recurrent vertigo attacks in the context of a pre-existing profound SNHL, and put DEH in the category of ipsilateral and contralateral DEH (14).

Although many vestibular tests have been applied in patients with DEH, including the electronystagmography (15), caloric test (15, 16), rotatory chair (15), and vestibular evoked myogenic potentials (VEMPs) (16, 17), no study examined vHIT or the relationship between caloric test and vHIT in these patients. Our study aimed to look into the features of these two VOR tests in DEH patients, and to explore their implications in the diagnosis of ELH or hydropic ear disease.

## Materials and Methods

### Study Population

A single-center retrospective study was conducted in the Department of Otorhinolaryngology, Union Hospital affiliated to Tongji Medical College, Huazhong University of Science and Technology, Wuhan, China.

Nineteen patients with DEH were diagnosed by experienced neurotologists against the diagnostic criteria formulated by the committee of the Japan Society for Equilibrium Research in 1987 (18): The ipsilateral DEH was diagnosed if (1) a prior event characterized by profound SNHL in one ear (precedent deafness); (2) delayed development of vertigo attacks without fluctuating hearing loss in the opposite ear; and (3) exclusion of central nervous system lesions, eighth nerve tumors, and other cochleovestibular diseases, such as syphilitic labyrinthitis. The diagnosis of the contralateral DEH was made if (1) a prior deafness in one ear; (2) delayed development of fluctuating hearing loss in the opposite ear that is sometimes associated with episodic attacks of vertigo; and (3) exclusion of central nervous system lesions, eighth nerve tumors, and other cochleovestibular diseases like syphilitic labyrinthitis. Profound SNHL was defined as a pure tone average of >90 dB over the 500, 1,000, and 2,000 Hz frequencies.

Exclusion criteria included: (1) middle or inner ear infections (otitis media, mastoiditis, labyrinthitis, etc.); (2) middle or inner ear anomaly (common cavity malformation, semicircular canal dysplasia, EVA, etc.); (3) having received previous ear surgery or intratympanic injections; (4) retro-cochlear lesions (vestibular schwannoma, internal acoustic canal stenosis, etc.); (5) head trauma; (6) systemic diseases; (7) disorders of central nervous system (vestibular migraine, multiple sclerosis, cerebellar infarction, etc); (8) having taken alcohol, caffeine or medications that would affect the results of vestibular tests (sedative, anti-depressant drugs, etc.) within 48 h before testing.

This study was conducted in strict accordance with the tenets of the Declaration of Helsinki. Informed consent was obtained from each patient and the project was approved by the ethical committee of Tongji Medical College of Huazhong University of Science and Technology, Wuhan, China.

### Methods

All patients with DEH during the interictal period underwent a battery of tests, including otoscopy, audiometry, videonystagmography, caloric test, horizontal vHIT in the same day. Non-contrast magnetic resonance imaging (MRI) was routinely performed to rule out middle or inner ear infection and malformation. If retro-cochlear lesion was suspected, the patient would receive contrast-enhanced MRI.

### Pure-Tone Audiometry

Pure-tone audiometry was conducted in a soundproof room, at 125, 250, 500, 1,000, 2,000, 4,000, and 8,000 Hz. Pure tone average (PTA) was calculated to be the simple arithmetic mean for frequencies of 500, 1,000, and 2,000 Hz.

### Videonystagmography

Vestibular tests were performed by using an infrared videonystagmography system (Visual Eyes VNG, Micromedical Technologies, Chatham, IL, USA) in accordance with a standard test protocol, including spontaneous nystagmus test, gaze test, saccades, smooth pursuit, and optokinetic test. The Dix-Hallpike and Roll tests were also videonystagmugraphically performed.

The bithermal caloric test was conducted by using infrared videonystagmography. The subject lay supine with their head and upper trunk elevated at 30°. Each ear was irrigated alternately with a constant flow of air, with the temperature for warm or cool stimulation set at 50 and 24°C, respectively. The duration of each caloric irrigation lasted 60 s. Upon each irrigation, the maximum slow phase velocity (SPV_max_) of caloric nystagmus was measured, and the canal paresis (CP) was calculated by using the traditional Jongkees' formula. In this study, if the asymmetry of the caloric nystagmus between the left and right ear was ≥25%, CP was considered significant in the horizontal SCC, indicating an abnormal caloric response. According to the published criteria (19), if the summated SPV_max_ of the nystagmus was <20°/s under four stimulation conditions, the caloric response is believed to indicate bilateral vestibular hypofunction. In this case, ice water irrigation (4°C, 1.0 ml) would be used to confirm the caloric unresponsiveness.

### Horizontal Video Head Impulse Testing

The horizontal SCC vHIT was conducted using an ICS Impulse system (GN Otometrics, Denmark) in accordance with the manufacturer's instructions by experienced technicians. Each subject wore a pair of lightweight, tightly-fitting goggles equipped with a small video oculography camera to record and analyze the eye movement. Patient was seated upright facing the wall 1.0 m away and was instructed to fixate a static target on the wall. The patient's head was passively and randomly rotated to the left and right with a low amplitude (5~15°) and at a high peak velocity (150~250°/s) in an abrupt, brief and unpredictable manner. At least 20 head impulses were delivered in each direction. Re-fixation saccades were categorized, in terms of their appearance, as covert and overt. If the velocity of the saccade exceeded 50°/s, they were deemed positive. In the present study, it was taken as abnormal if the horizontal vHIT gain < 0.8 and re-fixation saccades appeared.

## Results

### Clinical Characteristics

Nineteen DEH patients were included (12 females, 7 males; mean age, 38.9 year; range, 13–65 year). According to the diagnostic criteria, 17 patients were diagnosed with ipsilateral DEH and two as having contralateral DEH. All 19 patients reported precedent hearing loss and episodic vertigo attacks. Fifteen patients reported profound hearing loss of unknown etiology in early childhood. In three cases, hearing loss was caused by sudden SNHL in adulthood and in one case deafness was associated with meningitis in childhood (Table 1). A precise latency interval between the precedent hearing loss and the hydropic symptoms (recurrent vertigo or fluctuating hearing loss) could not be obtained, since the onset time of hearing loss was uncertain in most patients. The hydropic symptoms lasted 5.3 years on average (range: 0–25 years) before inclusion into this study. The duration of vertigo attacks lasted 20 min to 10 h. The frequency of vertigo attacks varied from 3 times a week to one time in several years. Other major complaints included: tinnitus and/or aural fullness (either persistent or fluctuating). Vestibular drop attack occurred in two cases (patient No. 4 and 9). Of note, in one case (patient No. 17) of ipsilateral DEH, concurrent horizontal SCC type BPPV was diagnosed in the opposite ear, and the patient suffered from both the Ménière -like episodic vertigo lasting for over 1 h during the past several months and recurrent transient positional vertigo while lying down or getting up before presentation. No migraine symptoms or history of migraine was recorded.

**Table 1:** The clinical features and audio-vestibular data in 19 patients with delayed endolymphatic hydrops.

**Patient no**.	**Age**	**Gender**	**Cause of deafness**	**Age at onset of deafness**	**Age at onset of vertigo**	**Side**	**Type**	**PTA**		**Caloric**		**Horizontal vHIT**
								**Right**	**Left**	**Response/weak side**	**CP (%)**	**Response/affected side**
1	65	M	Unknown	Early childhood	40	R	ipsil-	SO	26	A/R	43	Negative
2	31	M	SSHNL	28	29	R	ipsil-	93	5	A/R	36	Negative
3	30	M	Unknown	Early childhood	27	R	ipsil-	SO	8	A/R	36	Negative
4	20	F	Unknown	Early childhood	18	L	contra-	101	78	A/R	27	Positive/R
5	60	F	Unknown	Early childhood	50	L	contra-	SO	36	N	1	Negative
6	59	F	SSHNL	44	59	R	ipsil-	SO	15	A/R	29	Negative
7	44	F	Unknown	Early childhood	33	R	ipsil-	SO	15	N	22	Negative
8	13	F	Unknown	Early childhood	11	L	ipsil-	10	91	A/L	35	Negative
9	54	F	Unknown	Early childhood	54	R	ipsil-	SO	23	A/R	54	Negative
10	26	F	Unknown	Early childhood	22	R	ipsil-	SO	13	A/R	41	Negative
11	61	F	Unknown	Early childhood	47	L	ipsil-	23	SO	A/L	26	Negative
12	22	F	Unknown	Early childhood	22	L	ipsil-	10	SO	N	22	Negative
13	29	M	Unknown	Early childhood	26	R	ipsil-	106	11	N	3	Negative
14	25	F	Unknown	5	24	L	ipsil-	15	SO	A/L	85	Negative
15	16	M	Unknown	Early childhood	10	L	ipsil-	5	105	A/L	100	Positive/L
16	58	F	Unknown	Early childhood	51	R	ipsil-	98	16	A/R	100	Negative
17	56	M	Meningitis	Early childhood	56	L	ipsil-	18	SO	A/L	84	Negative
18	56	F	SSHNL	40	46	L	ipsil-	13	SO	N	19	Negative
19	15	M	Unknown	6	15	L	ipsil-	15	100	A/R	100	Positive/R

*PTA, pure tone average, which was established as the simple arithmetic mean for frequencies of 500, 1,000, and 2,000 Hz; Ipsil-, ipsilateral type; Contra-, contralateral type; CP, canal paresis; R, right side; L, left side; SO, scale out; A, abnormal; N, normal. SSNHL, sudden sensorineural hearing loss*.

### Audio-Vestibular Evaluations

Audio-vestibular test results are presented in Table 1. All 19 patients had profound SNHL (>90 dB) in one ear. For the better-hearing ear, the PTA of 500, 1,000, and 2,000 Hz was <26 dB in 16 ears, 26–40 dB in two ears and >40 dB in 1 ear. Spontaneous nystagmus was detected in five out of 19 patients (26.3%). Among these five cases, four cases were of ipsilateral DEH type with spontaneous nystagmus beating to the affected side in one case and to the non-affected side in the other three cases, and one case was of contralateral DEH type with spontaneous nystagmus beating to the affected side. No pathological results were observed in the gaze, saccades, smooth pursuit, and optokinetic test.

In this series, 14 (73.7%) had reduced caloric response, and only three cases (15.8%) had abnormal horizontal vHIT responses. Of the 17 patients with ipsilateral DEH, abnormal CP was observed in 76.5% (13/17) of the patients and 11.8% (2/17) yielded abnormal vHIT result. In 12 cases, abnormal CP was ipsilateral in the ear with preexisting hearing loss and in one patient abnormal CP was in the opposite ear. For the two patients with both abnormal CP and vHIT results, abnormal response occurred in the previously damaged ear in one case, and in the opposite ear in the other case. Of the two patients with contralateral DEH, abnormal CP and vHIT were detected in one case (50%), and abnormalities occurred in the ear with preexisting hearing loss. For all subjects, MRI did not reveal any retro-cochlear lesion.

### Treatment and Following-Up

Lifestyle modification was recommended to the patients, including low-sodium diet, low caffeine consumption, and abstinence from stimulants. In all 19 patients, initial medication included the betahistine or diuretics, lasting for at least 3–6 months. Two patients (No. 9 and 14) received one course of intratympanic dexamesone due to unsatisfactory vertigo control. No ablative treatment was administered in this series, especially for the contralateral type of DEH. A follow-up, lasting 3 months−3.0 years, showed that, against the guidelines for MD proposed by American Academy of Otolaryngology-Head and Neck Surgery in 1995 (20), complete or substantial vertigo control (class A or B) was achieved in 16 cases and limited control (class C) in three cases. For the patient with concomitant horizontal SCC type BPPV, Gufoni maneuver successfully cured the positional vertigo.

## Discussion

In this study, we investigated the function of horizontal SCC VOR in patients with DEH. In our series, 73.7% (14/19) of the DEH patients showed abnormal caloric response. Meanwhile, only 15.8% (3/19) of the patients yielded abnormal horizontal vHIT results. To the best of our knowledge, this was the first study which examined the VOR function measured by vHIT and identified a discrepancy between the results of vHIT and the caloric test in patients with DEH.

Until now, two hypotheses have been put forward to explain this dissociation. First, the hydrostatic temperature dissipation hypothesis attributed the discrepancy to the different test mechanisms between caloric test and vHIT. The distended membranous duct of the horizontal SCC has been suggested to permit convective recirculation within the duct, leading to a diminished thermally- induced pressure gradient across the cupula and thus to less deflection of the cupula and hair cells and less caloric nystagmus. This assumption is supported by the observation that, in MD patients with normal vHIT results, the reduced caloric response was correlated with the severity of vestibular hydrops (as demonstrated by intravenous gadolinium-enhanced MRI of the inner-ear) (21). Second, the dual frequency hypothesis proposes that a differential activation of vestibular hair cells by stimuli with different frequencies might be responsible for the dissociation. It is believed that type II hair cells are sensitive to the low-frequency (caloric) stimulus and the type I hair cells to the high-frequency (head impulse) stimulus. Previous histological studies showed that damage was more severe in type II hair cells than in type I hair cells as MD deteriorates. This theory is recently being challenged by multiple findings (22–24). In addition, the effect of central compensation cannot be fully ruled out. Since rapid angular head movements are frequent in everyday activities, the response to rapid head movement may adapt better to vestibular hypofunction than the non-physiological caloric response.

Recently, multiple studies have demonstrated this disagreement in patients with MD (idiopathic ELH). McCaslin et al. (25) and McGarvie et al. (6) reported normal vHIT response but abnormal caloric results in patients with definite MD, respectively. A recent study by Rubin et al. (7) found that the caloric test was abnormal in 34 out of 37 MD patients, yet vHIT yielded normal gain. Jung et al. demonstrated that, apart from the patients with MD, this dissociation also occurred in patients with EVA (2). Abnormal CP was detected in four out of 10 cases and abnormal vHIT response was found only in one case. In contrast, all 19 patients with vestibular neuritis exhibited abnormal CP and 18 of them had abnormal vHIT response. Similarly, this discrepancy was also observed in patients with congenital semicircular canal malformation (common cavity formed by the vestibule and horizontal SCC), thus further supporting the dissipation theory (26). By retrospectively analyzing the conditions that show such dissociation in a non-homogenous group of patients complaining of vertigo or imbalance, Hannigan et al. (5) found that MD subjects comprised 75% (27/36) of the dissociation group. Of the 73 MD patients tested, 27 showed a dissociation and, of the 533 non-MD subjects tested, only nine showed such dissociation. Since MRI-demonstratable ELH is the common imaging feature of DEH, MD, EVA, and common cavity malformation (27), we suggest that this dissociation between the caloric test and vHIT might be a distinctive pattern of vestibular deficit in hydropic ear disease (28).

In this study, we showed that, in patients with ipsilateral DEH, 70.6% (12/17) had abnormal CP in the ear with previous cochlear damage and 5.8% (1/17) had abnormal CP in the opposite ear. Our result was in agreement with that reported by Schuknecht et al. who found that 80% of the ipsilateral DEH patients had unilateral caloric weakness in the hydropic ear and 9% had such hypofunction in the opposite ear (29). The substantially higher incidence of abnormal CP in the ear with precedent hearing loss than in the contralateral ear might be ascribed to the fact that: (1) bilateral ELH is commonly present in ipsilateral DEH patients, as demonstrated by gadolinium-enhanced MRI (30, 31), and (2) the area of ELH in the deaf ear was significantly larger than or practically equivalent to that of the better-hearing ear (31).

In the present study, 50% (1/2) of the patients with contralateral DEH showed the abnormal CP and vHIT in the previously deaf ear rather than the opposite ear. The pattern of ELH distribution and vestibular deficit is rather complicated in contralateral DEH, indicating that its pathology is more involved compared to its ipsilateral counterpart (30, 32, 33). Further studies with larger sample sizes are warranted to elucidate the pathogenesis.

We found a vHIT deficit in 15.8% and a caloric weakness in 73.7% of our DEH patients. No other studies investigated the VOR function at these high frequencies in patients with this condition. Alternatively, rotatory chair test can assess the VOR system across the low to middle frequencies (0.01–1.00 Hz). To date, only one published study has investigated the performance of rotary chair and caloric test in DEH patients and revealed that rotatory chair test identified vestibular hypofunction in 3% while caloric test detected the hypofunction in 59% of their patients (15). More evidence is needed to verify our results.

In hydropic ear disease with fluctuating nature, the caloric response was speculated to reflect the severity of ELH more than the impairment of VOR function. Clinically, the functional assessment or therapeutic decision may be biased if based on the caloric test alone. This was in agreement of Bodmer et al. (34) and Lin et al. (35), who challenge the functional significance of CP values or caloric unresponsiveness in the prediction of long-term vertigo control, while Hone et al. found an absence of ice water response was highly predictive of adequate vertigo control and recommend a total chemical ablation of VOR function by intratympanic gentamicin treatment (36). On the contrary, vHIT allows for an objective evaluation of the actual status of angular VOR pathways by using physiological stimuli and can give clinicians a dynamic and real-time picture of the VOR function. Previous studies have proven the value of vHIT in reflecting functional fluctuation, therapeutic endpoint and vertigo recurrence in MD patients (22, 35, 37, 38). Therefore, we suggest that vHIT is an objective screening test of choice for dynamically monitoring the VOR function in patients with hydropic ear disease. This is especially necessary for those receiving ablative therapy, in whom the effective vestibular impairment should be clearly defined and the progress of functional recovery should be closely monitored.

The study had several limitations. First, the sample size was small, especially for the contralateral cases, because DEH is not a common vestibular disorder. Second, we did not perform gadolinium-enhanced MRI, which might provide further imaging evidence to account for the findings of our VOR tests. Third, this study did not perform the rotatory chair test, in which the VOR time constant has been shown to provide the most reliable assay screening fixed peripheral vestibular loss (39). Since caloric-vHIT dissociation also occurs in fixed peripheral vestibular loss, for example, during the recovery phase of vestibular neuritis (40), supplement of rotary chair test would be helpful in excluding fixed peripheral vestibular loss caused by ELH. In future, large-scale studies involving comprehensive audio-vestibular tests and contrast-enhanced MRI examinations are warranted to further understand the pathophysiology of DEH.

## Conclusion

Abnormal caloric response in the presence of a preserved vHIT might be a distinctive pattern of vestibular deficit in patients with DEH, a rare variant of Ménière's disease. More evidence is needed to clarify the clinical implication of this dissociation in hydropic ear disease.

## Data Availability Statement

The datasets generated for this study are available on request to the corresponding author.

## Ethics Statement

The studies involving human participants were reviewed and approved by Ethical committee of Tongji Medical College of Huazhong University of Science and Technology, Wuhan, China. Written informed consent to participate in this study was provided by the participants' legal guardian/next of kin.

## Author Contributions

BL: study conception and design, data acquisition, and critical review of the manuscript. YL: data analysis and interpretation, drafting and revision of the manuscript.

## Conflict of Interest

The authors declare that the research was conducted in the absence of any commercial or financial relationships that could be construed as a potential conflict of interest.
